# Exopolysaccharide Produced by *Pediococcus pentosaceus* E8: Structure, Bio-Activities, and Its Potential Application

**DOI:** 10.3389/fmicb.2022.923522

**Published:** 2022-06-22

**Authors:** Guangyang Jiang, Juan He, Longzhan Gan, Xiaoguang Li, Zhe Xu, Li Yang, Ran Li, Yongqiang Tian

**Affiliations:** ^1^College of Biomass Science and Engineering, Sichuan University, Chengdu, China; ^2^Key Laboratory of Leather Chemistry and Engineering, Ministry of Education, Sichuan University, Chengdu, China; ^3^Key Laboratory of Bio-Resources and Eco-Environment of Ministry of Education, College of Life Sciences, Sichuan University, Chengdu, China; ^4^Department of Microbiology, Faculty of Agriculture and Forestry, University of Helsinki, Helsinki, Finland

**Keywords:** exopolysaccharide, structural analysis, emulsifying activity, strawberry preservation, *Pediococcus pentosaceus*

## Abstract

The novel exopolysaccharide EPS-E8, secreted by *Pediococcus pentosaceus* E8, was obtained by anion-exchange and gel filtration chromatography. Structural analyses identified EPS-E8 as a heteropolysaccharide containing mannose, glucose, and galactose. Its major backbone consists of →2)-α-D-Man*p*-(1→2,6)-α-D-Glc*p*-(1→6)-α-D-Man*p*-(1→, and its molecular weight is 5.02 × 10^4^ g/mol. Using atomic force microscopy and scanning electron microscopy, many spherical and irregular reticular-like shapes were observed in the microstructure of EPS-E8. EPS-E8 has outstanding thermal stability (305.7°C). Both the zeta potential absolute value and average particle diameter increased gradually with increasing concentration. Moreover, at a concentration of 10 mg/ml, the antioxidant capacities of, 1-Diphenyl-2-picrylhydrazyl (DPPH), ABTS and hydroxyl radical were 50.62 ± 0.5%, 52.17 ± 1.4%, and 58.91 ± 0.7%, respectively. EPS-E8 possesses excellent emulsifying properties against several food-grade oils, and its activity is retained under various conditions (temperature, pH, and ionic strength). Finally, we found that EPS-E8 as a polysaccharide-based coating could reduce the weight loss and malondialdehyde (MDA) content of strawberry, as well as preserving the vitamin C and soluble solid content during storage at 20°C. Together, the results support the potential application of EPS-E8 as an emulsifier, and a polysaccharide-based coating in fruit preservation.

## Graphical Abstract

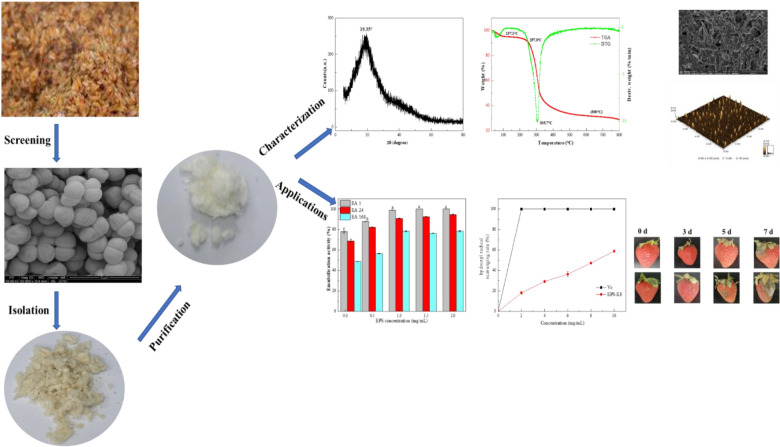

## Highlights

- An exopolysaccharide (EPS), purified from *Pediococcus pentosaceus* E8, showed noteworthy thermal stability under high temperature.
- EPS-E8 had unique backbone structure of →2)-α-D-Man*p*-(1→2,6)-α-D-Glc*p*-(1→6)-α-D-Man*p*-(1→.
- EPS-E8 exhibited excellent emulsifying activity and potent antioxidant activities.
- EPS-E8 as a coating could effectively delay the senescence of strawberries.
- EPS-E8 may be exploitable to improve the shelf-life of strawberries.

## Introduction

As biologically synthesized macromolecules, exopolysaccharides (EPSs) are loosely attached to the bacterial cell wall or dispersed into their surroundings during the growth phase of microorganisms (Bai et al., 2021; Buksa et al., 2021). Depending on the diversity of monosaccharide subunits and their biosynthesis pathways, EPSs are classified into two types: homopolysaccharides, which contain repeats of one type of monosaccharide, and heteropolysaccharides, which contain two or more monosaccharides. As representatives of multifunctional natural polymers, EPSs possess an evident advantage over polysaccharides produced by animal and plant cells because they do not rely on external environmental conditions (e.g., season or geography). Among EPS-producing bacteria, lactic acid bacteria (LAB) stand out because of their status as generally recognized as safe (GRAS), and EPSs produced by LAB (LAB-EPSs) are considered as safe agents (Rajoka et al., 2020). In previous literature, a substantial number of EPS-producing LAB have been isolated from traditional Chinese fermented foods. Prominent examples are *Enterococcus* sp. F2, which has been isolated from fermented soy beans (Jiang et al., 2021), *Lactobacillus plantarum* HY, which has been isolated from pickle (Liu et al., 2019), and *Lactobacillus plantarum* YW11, which has been isolated from kefir (Wang et al., 2015). In addition to the isolation of EPS-producing LAB, studies have also clarified the potential bioactivities of LAB-EPSs, such as antioxidant, anti-cancer, immunomodulatory, and cholesterol-lowering activities (Saadat et al., 2019). Because of the unique health benefits and physicochemical properties, LAB-EPSs have been widely used in several fields, e.g., in the food additive and active edible packaging industry, and for adsorption treatment of heavy metal from wastewater. Recently, finding and characterizing novel EPSs with bioactivities and improved functional properties has drawn increasing interest, and EPSs from LAB are a particular focus. Structural characterizations, physiological activities, and functional properties of EPS secreted by *Pediococcus pentosaceus* have been rarely reported. Ayyash et al. (2020a) found that EPS isolated from *P. pentosaceus* M41 exhibited noteworthy antitumor, antioxidant, antidiabetic, and antibacterial activities. Bai et al. (2021) reported that EPS secreted by *P. acidilactici* MT41-11 could be a potential antioxidant agent, and a prebiotic substance added to food. *P. pentosaceus* is a Gram-positive, facultatively anaerobic, non-motile, and non-spore-forming species, that is crucial in the fermentation of Chinese cereal vinegar (Gong et al., 2021). To date, *P. pentosaceus* has not been isolated from Chinese cereal vinegar and its EPS-producing properties has not been characterized.

Due to the film-forming ability, EPSs can be applied as protective coatings in some foods. Moreover, EPSs own some healthy benefits and thus are distributed as bio-functional agents in the food and pharmaceutical industry. In addition, it has been found that these polysaccharide-based coatings can effectively preserve fruits due to their antioxidant activity (Guerreiro et al., 2015). For instance, Gol et al. (2015) found that polysaccharide-based coatings positively influenced soluble solid content and pH of Jamun fruit, and significant delayed the weight loss. Yuan et al. (2020) reported that EPSs from *Pythium arrhenomanes* as a polysaccharide-based coating could be helpful prolonging the shelf-life of strawberries. Strawberry is one of the most fragile and perishable fruits, vulnerable to mechanical damage, physiological degradation, water loss and fungal decay, which makes the preservation of strawberries problematic (Matar et al., 2018).

In the present work, a new type of EPS-producing strain, *P. pentosaceus* E8, was screened from cereal vinegar. The chemical structure of the EPS produced by *P. pentosaceus* E8 was characterized by a series of analytical methods: ultraviolet-visible spectroscopy (UV-vis), high-performance liquid chromatography (HPLC), Fourier transform-infrared spectroscopy (FT-IR), nuclear magnetic resonance spectroscopy (NMR), thermogram analysis (TGA), differential scanning calorimeter (DSC), and scanning electron microscopy (SEM). Furthermore, the bioactive and emulsifying properties of the novel EPS from *P. pentosaceus* E8 have been assessed. Additionally, we explored the potential of this EPS to be utilized as a coating in the strawberry preservation.

## Materials and Methods

### Screening of Strains and 16S rDNA Sequencing

Cereal vinegar samples (provided by Qianhe Food Co., Ltd., Meishan, China) were uniformly dispersed and then continuously diluted in sterile deionized water. Treated samples were cultured on de Man, Rogosa, and Sharpe (MRS) medium and incubated at 30°C to obtain a single colony. Colonies were picked and bacterial species were identified based on morphology and physiological characteristics coupled with 16S rDNA sequencing. Universal primers pairs 27F (5’-AGAGTTTGATCCTGGCTCAG-3’) and 1492R (5’-GGTTACCTTGTTACGACTT-3’) were obtained from Tsingke Biological Technology (Sichuan, China) and were used for PCR and sequencing. The phylogenetic tree was established using the neighbor-joining method by MEGA 7.0. The *P. pentosaceus* strain with the highest EPS yield of 1.37 ± 0.81 g/L was named E8 and used for the further production and characterization of EPSs.

### Isolation and Purification of Exopolysaccharides

*Pediococcus pentosaceus* E8 was inoculated into MRS containing 4% sucrose and incubated for 48 h; then, crude EPS was isolated according to a previously reported method (Ayyash et al., 2020a). In short, the cell-free supernatant was first collected by centrifugation (10,000 × *g*, 15 min, 4 °C) after fermentation. Then, three volumes of pre-chilled 95% (v/v) ethanol were added, and the mixture was incubated overnight at 4°C to precipitate crude EPSs. After that, the precipitate was dissolved in ultrapure water as crude EPS. To remove proteins, crude EPSs were treated with papain (800 U/mL, pH 6.0) combined with Sevage reagent (chloroform/*n*-butanol at 4:1, v/v) (Gan et al., 2020a). Before chromatographic analysis, a dialysis bag (cut-off 14 kDa, Yibo Biological Technology Co., Ltd., Hunan, China) was used to dialyze the treated crude EPSs against distilled-deionized water for 3 days and the dialyzed sample was then lyophilized. The lyophilized EPSs sample was redissolved and sequentially separated by a DEAE-52 anion-exchange chromatographic column (2.6 × 20 cm; GE Healthcare, Gothenburg, Sweden) with different concentrations NaCl solution (0, 0.1, 0.3, and 0.5 M) at a flow rate of 2 mL/min. Then, the mixture was eluted with ultrapure water (1 mL/min) via Sephadex S-300 HR chromatography (1.6 × 100 cm; GE Healthcare, Gothenburg, Sweden). Finally, the major fraction of crude EPSs obtained from *P. pentosaceus* E8, designated EPS-E8, was combined and lyophilized for further analysis of structure and bio-functional activities.

### Chemical Composition Assays

The total sugar and protein contents were measured by using the phenol-sulfuric acid colorimetric (Bradford, 1976) and Bradford methods (DuBois et al., 2002), respectively. The UV spectrum of the EPS-E8 solution (1 mg/mL) was measured via a U-3900H spectrophotometer (Hitachi, Japan) by scanning from 200 to 800 nm to test for the presence of protein or nucleic acids.

### Molecular Weight and Monosaccharide Composition Analysis

To determine the homogeneity and average molecular weight of EPS-E8, high-performance size-exclusion chromatography (HPSEC) and multi-angle laser light scattering (MALLS) spectrometry (DAWN HELEOS II, Wyatt Technology, CA, United States) were used, respectively. Ten milligrams of EPS-E8 sample were dissolved in 0.1 mol/L NaNO_3_ solution (1 mL) and filtered through a 0.22-μm filter (Xinya Purification Equipment Co., Ltd., Shanghai, China). After that, the EPS-E8 solution (10 mg/mL, 100 μL) was loaded onto separation system with different columns (Shodex Ohpak SB-805, 804, and 803, Shodex, Tokyo, Japan). Separation conditions were: 0.1 mol/L NaNO_3_ solution as mobile phase, 0.4 mL/min, 60 °C. The data were acquired by ASTRA6.1 software (Wyatt Technology, CA, United States). Monosaccharide analysis of EPS-E8 was conducted by high-performance anion-exchange chromatography with pulsed amperometric detection (HPAEC-PAD). Before measurement, 5 mg of EPS-E8 were mixed with 1 mL of trifluoroacetic acid (TFA, 2.0 M) in a sealed glass ampoule and heated at 121 °C for 2 h, then dried by nitrogen blowing. The residue after drying was redissolved in deionized water and filtered through a 0.22-μm film. The EPS-E8 hydrolysate and standards (including fucose, rhamnose, arabinose, galactose, glucose, xylose, mannose, fructose, ribose, galacturonic acid, glucuronic acid, mannuronic acid, and guluronic acid) were further analyzed using a Dionex ICS-5000 ion chromatography system (Thermo Scientific, MA, United States) equipped with a Dionex CarboPac PA-20 analytical column and a Dionex ED50A electrochemical detector.

### Methylation Analysis of EPS-E8

Methylation analysis was performed according to a previously described method (Gan et al., 2020a). Briefly, 5 mL DMSO and 10 mg NaOH were used to dissolve the freeze-dried EPS-E8 (10 mg); then, 0.5 mL methyl iodide was added for the reaction. The obtained product was separated with CH_2_Cl_2_, washed 3–5 times, and dried by nitrogen blowing. The complete methylation product was further hydrolyzed by 2.0 M TFA at 121°C for 2 h, and then reduced with sodium borodeuteride and acetylated with acetic anhydride (1:1, v/v) at 100°C for 2.5 h. The obtained partially methylated alditol acetates were analyzed using a gas chromatography-mass spectrometer (GC-MS; 6890A-5975C, Agilent Technologies, Palo Alto, United States) equipped with an HP-5MS capillary column.

### Spectroscopic Analysis by Fourier Transform-Infrared Spectroscopy and Nuclear Magnetic Resonance Spectroscopy

The main functional groups of EPS-E8 were analyzed on a Nicolet iS1S0 infrared spectrometer (Thermo Nicolet Inc., WI, United States) at a frequency range of 500–4000 cm^–1^ (Jiang et al., 2020). A total of 40 mg of dry EPS-E8 sample was exchanged with deuterium by lyophilizing against deuterium oxide (D_2_O) twice and dissolved in D_2_O (99.9% D) before NMR analysis. 1D NMR (1H- and 13C NMR) and 2D NMR (COSY, TOCSY, NOESY, HSQC, and HMBC) were recorded on a Bruker 600 MHZ spectrometer (Bruker, Karlsruhe, Switzerland).

### Thermal Properties and X-ray Diffraction

The thermal properties were assessed by TGA, differential thermal analysis (DTG), and DSC (NETZSCH, Free State of Bavaria, Germany). TGA and DTG were recorded from a temperature of 35 to 800 °C. X-ray diffraction (XRD) data of EPS was obtained on a D8 advance X-ray diffractometer (Bruker, Karlsruhe, Switzerland), under running conditions of 40 mA, 40 kV, an angular range of 5° and 80°, and a steep of 0.02°/min.

### Zeta Potential, Particle Size, Scanning Electron Microscopy, and Atomic Force Microscopy Examination

A ZEN5600 Zetasizer NanoPlus (Malvern Instruments, Malvern, United Kingdom) was used to examine the corresponding properties of EPS-E8 (1–5% w/v) at 25°C. The microscopic morphology of EPS-E8 was characterized via SEM (Apreo 2C, Thermo Scientific, MA, United States) at an accelerating voltage of 15 kV. The molecular morphology of EPS-E8 was identified using atomic force microscopy (AFM; SPM-9600, Shimadzu, Japan).

### Emulsifying Properties of EPS-E8

The emulsifying property of the EPS-E8 sample was assessed according to a previously reported method (Maalej et al., 2016). A total of 6 mL of various edible oils (olive oil, coconut oil, peanut oil, sunflower oil, soybean oil, palm oil, rap oil, and soybean oil) and hydrocarbons (*n*-hexane and *n*-octane) were mixed with the EPS-E8 sample solution (1 mg/mL, 4 mL) and stirred for 5 min. Various emulsifying activities (EA, %), namely EA 1, EA 24, and EA 168, were recorded after 1, 24, and 168 h, respectively. Then, olive oil was utilized as testing oil to assess the influence of the EPS-E8 concentration on its emulsifying activity. The emulsions obtained with olive oil were investigated with different EPS-E8 concentrations (0–2%) for evaluating EA 1, EA 24, and EA 168. Moreover, the effects of temperature (25–100°C), pH (4.0–12.0), and ionic strength (0–2.0 M NaCl) on emulsion stability were also evaluated. EA was calculated as follows:

EA (%) = (emulsion layer height/mixture overall height) × 100

### Analysis of Antioxidant Property

#### 1,1-Diphenyl-2-picrylhydrazyl Radical Scavenging Ability

The 1,1-Diphenyl-2-picrylhydrazyl (DPPH) radical scavenging ability of EPS-E8 was assessed according to a previously published method (Teng et al., 2021). Vitamin C (VC) was used as positive control. The DPPH radical scavenging activity was calculated according to the following formula:


$$\begin{array}{c} \mathrm{Scavenging}\mathrm{activity}\left(\%\right)=1-\\ {}[(\mathrm{absorbance}\mathrm{of}\mathrm{sample}\mathrm{mixed}\mathrm{with}\mathrm{DPPH}\mathrm{solution}-\\ \mathrm{absorbance}\mathrm{of}\mathrm{sample})/\mathrm{absorbance}\mathrm{of}\mathrm{DPPH}\mathrm{solution}]\times 100 \end{array}$$

#### ABTS^+^ Free Radical Scavenging Ability

The ABTS radical scavenging activity of EPS-E8 was determined using a previously reported method (Gu et al., 2020). The absorbance of sample and control at 734 nm were recorded and then calculated using the formula:


$$\begin{array}{c} \mathrm{Scavenging}\mathrm{capacity}\left(\%\right)=1-(\mathrm{absorbance}\mathrm{of}\mathrm{sample}/\\ \mathrm{absorbance}\mathrm{of}\mathrm{control})\times 100 \end{array}$$

#### Hydroxyl Free Radical Scavenging Ability

The hydroxyl radical scavenging activity of EPS was determined based on a previously reported method (Gu et al., 2020). The absorbance of the resulting mixture was measured at 510 nm. The scavenging capacity was calculated as follows:

Scavenging capacity (%) = 1 – [(absorbance of sample – absorbance of reagent blank)/absorbance of control] × 100

### Application of Exopolysaccharide (EPS) From *Pediococcus pentosaceus* E8 in Strawberry Preservation

#### Treatment of Fruit

Fresh strawberries exhibiting analogous size, shape, and ripeness and without visual defects were purchased from the local market (Chengdu, Sichuan, China). All strawberries were divided into two groups: group A was dipped in 8% EPS-E8 solution for 5 min, air-dried, and then incubated at 20 ± 1°C for 7 days; group B was used as control, strawberries were dipped in ultrapure water and manipulated in the same way as group A. The preservation indexes were analyzed every 24 h for each group.

#### Determination of Weight Loss Ratio and Soluble Solids Content

The weight of strawberries was weighed every day via the method described by Yan et al. (2020). The weight loss of strawberries was monitored using an analytical balance by weight determination on each day to calculate the weight loss ratio by the following:


$$\begin{array}{c} \mathrm{weight}\mathrm{loss}\mathrm{rate}\left(\%\right)=[(\mathrm{the}\mathrm{initial}\mathrm{weight}-\mathrm{the}\mathrm{sample}\\ \mathrm{weight}\mathrm{after}\mathrm{storage}\mathrm{time})/\mathrm{the}\mathrm{initial}\mathrm{weight}]\times 100 \end{array}$$

A PAL-1 digital refractometer (ATAGO Co. Ltd., Tokyo, Japan) was used to determine the soluble solid contents of strawberries.

#### Determination of Malondialdehyde Content

To determine the Malondialdehyde (MDA) content, a slightly modified method from Yuan et al. (2020) was applied. Briefly, strawberry sample (1 g) was thoroughly ground in 5 mL of 5% trichloroacetic acid (TCA) and centrifuged at 10,000 × *g* for 10 min at 4°C. Subsequently, the supernatant was mixed with 0.67% thiobarbituric acid (2 mL), and sequentially transferred to a water bath (100°C, 20 min) and an ice bath (0°C, 5 min). The treated mixture was finally centrifuged (10,000 × *g*, 4°C, 15 min), and supernatant was collected to measure its absorbance value at 450 nm (*A*450), 532 nm (*A*532), and 600 nm (*A*600). The MDA content was calculated according to following formula:


MDAcontent=6.45×(A532-A600)-0.56×A450

#### Determination of Vitamin C Content

Vitamin C (VC) content was measured based on the method of Wu et al. (2015) with minor modifications. Briefly, 1 g of strawberry sample was ground with 5% TCA (5 mL) and then centrifuged at 10,000 × *g* (10 min, 4 °C). Afterwards, 1 mL of 5% TCA, absolute alcohol, 0.4% phosphoric acid, 0.5% 1,10-phenanthroline, and 0.03% ferric chloride were added to the supernatant. The mixture was maintained at 30 °C for 30 min, and then the absorbance value of the mixture was recorded at 510 nm. The VC content was calculated with the established standard curve:


$$Y=0.0186X+0.0084\left(R^{2}=0.9994\right)$$

where X is VC content, Y is absorbance value at 510 nm.

### Statistical Analyses

All measurements were carried out in triplicates, and the data are expressed as means ± standard deviations and analyzed for variance by SPSS 22.0 (IBM, Armonk, NY, United States), using ANOVA followed by Duncan’s multiple-range test.

## Results and Discussion

### Identification of Selected Strain

**FIGURE 1:**
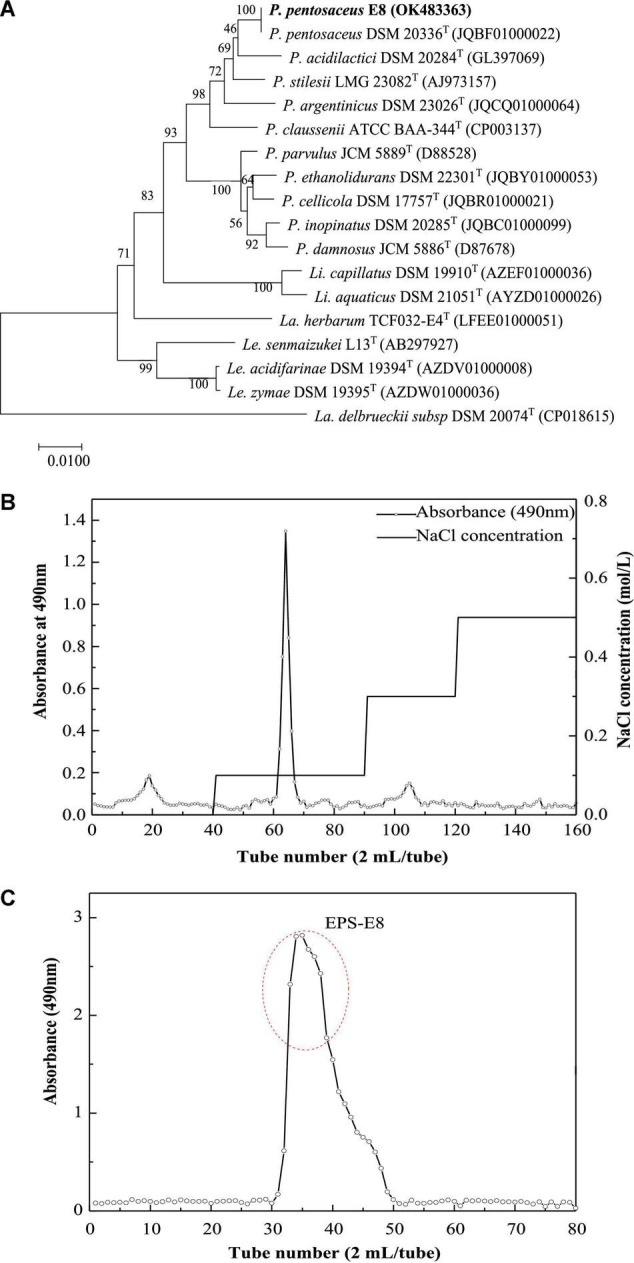
**(A)** Neighbor-joining tree based on 16S rDNA sequences showing genetic relatedness between *Pediococcus pentosaceus* E8 and related species; **(B)** DEAE-52 anion-exchange chromatogram; **(C)** Sephacryl S-300 HR chromatographic profile.

The EPS-producing strain, named E8, was isolated from cereal vinegar. Under the SEM, strain E8 was either found in pairs or short chains, which is the typical morphology of *Pediococcus* (Supplementary Figure 1). According to the 16S rDNA sequence of strain E8, a phylogenetic tree was constructed by the neighbor-joining method. The phylogenetic tree (Figure 1A) showed that strain E8 clustered with *P. pentosaceus* DSM 20336T (GenBank accession number: JQBF01000022). Thus, the identified strain was named *Pediococcus pentosaceus* E8. The 16S rDNA sequence of *P. pentosaceus* E8 was uploaded to the GenBank database under the accession number OK483363.

### Exopolysaccharide Production and Purification

**FIGURE 2:**
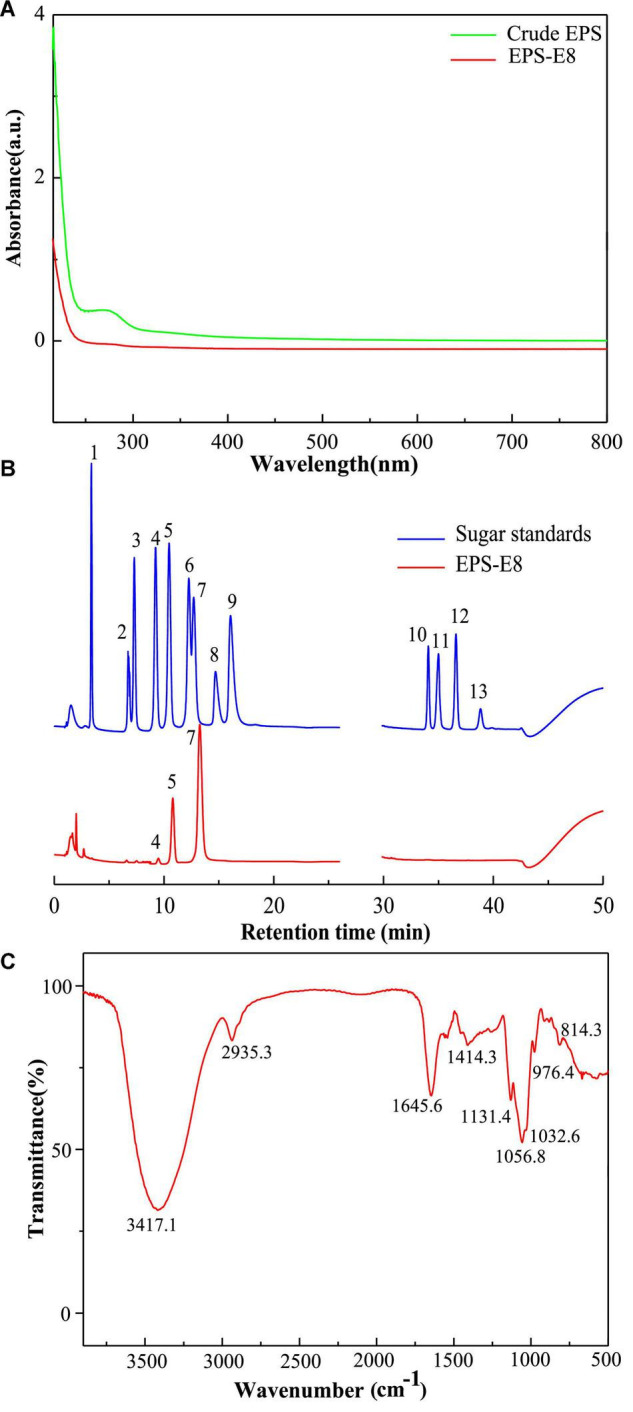
**(A)** UV–vis absorption spectrum of the purified EPS-E8 sample; **(B)** HPAEC-PAD profiles of monosaccharide standards (blue curve, peak identities:1, fucose; 2, rhamnose; 3, arabinose; 4, galactose; 5, glucose; 6, xylose; 7, mannose; 8, fructose; 9, ribose; 10, galacturonic acid; 11, guluronic acid; 12, glucuronic acid; 13, mannuronic acid) and EPS-E8 (red curve); **(C)** FTIR spectrum of EPS-E8 sample.

The crude EPSs of *P. pentosaceus* E8 were harvested via fermentation, ethanol precipitation, deproteinization, and dialysis. Crude EPSs were first purified by a DEAE-52 anion exchange chromatography column, and one major peak and two minor peaks were observed (Figure 1B). Next, the fractions from the major peak were collected and further separated using a Sephadex S-300 HR gel-filtration column. As shown in Figure 1C, a single peak was obtained, named EPS-E8. Finally, the fractions of EPS-E8 were collected and lyophilized to represent the purified polysaccharide. As tabulated in Table 1, the total sugar concentration of EPS-E8 was 97.9 ± 1.8%, whereas proteins and sulfates were not found in EPS-E8. The total sugar concentration of EPS-E8 was significantly higher than that of EPS obtained from *L. helveticus* LZ-R-5 (94.35 ± 1.03%) (You et al., 2020) and *L. plantarum* RS20D (84.21%) (Zhu et al., 2019). Moreover, the UV-vis spectrum of EPS-E8 showed no apparent peaks at 280 or 260 nm, further confirming that proteins and nucleic acids had been fully removed (Figure 2A).

**TABLE 1:** The physicochemical properties of the purified EPS-E8.

Physicochemical properties	
Total carbohydrate	97.9 ± 1.8%
Protein	Nd*
Sulfates	Nd*
**Relevant molecular parameters of the purified EPS-E8**	
Mw (Da)	5.02 × 10^4^
**Monosaccharide composition/relative content (%)**	
Mannose	80.39
Glucose	18.12
Galactose	1.49

*Nd, not detected.

### Molecular Weight and Monosaccharide Composition

Multi-angle laser light scattering and refractive index detection were conducted to determine the molecular weight of EPS-E8 using HPSEC-MALLS-RI (Table 1). The molecular weight (Mw) of EPS-E8 is 5.02 × 10^4^ g/mol, which is in line with the molecular weight range of LAB exopolysaccharides (10^4^–10^6^ g/mol) (Lynch et al., 2018). However, the Mw of EPS-E8 is lower than the Mw of EPS produced by *P. pentosaceus* M41 (6.82 × 10^5^ g/mol) (Ayyash et al., 2020a) and *L. plantarum* C70 (3.8 × 10^5^ g/mol) (Ayyash et al., 2020b), and higher than the Mw of EPS from *Bacillus* sp. S-1 (1.76 × 10^4^ g/mol) (Hu et al., 2019). These differences in Mw may be the result of inter-species and inter-strain variations. Generally, better water solubility and expanded chain conformation are observed in polysaccharides with low molecular weight, resulting in better bioactivities. The monosaccharide composition of EPS-E8 was authenticated using HPAEC-PAD technique. As illustrated in Figure 2B and Table 1, EPS-E8 mainly consists of mannose, glucose, and galactose at a molar ratio of 80.39: 18.12: 1.49. Interestingly, uronic acid was not detected in EPS-E8. In addition, mannose and glucose were predominant sugar ingredients in EPS-E8, accounting for 98.5% of all monosaccharides. According to the monosaccharide composition of EPS-E8, it can be concluded that it was a neutral heteropolysaccharide. The monosaccharide composition of EPS-E8 differed from *P. pentosaceus* M41 (arabinose, mannose, glucose, and galactose) (Ayyash et al., 2020a) and *P. pentosaceus* DPS (glucose, mannose, and fructose) (Abid et al., 2018). These differences may be ascribed to the influence of strains, media ingredients, and environmental conditions.

### Functional Groups and Glycosidic Linkages

FT-IR spectroscopy was used to analyze the main functional groups and chemical bonds of EPS-E8, the results of which are shown in Figure 2C. The strong and broad peak at 3417.1 cm^–1^ corresponds to the hydroxyl (OH) stretching vibration. The absorption band that appeared at 2935.3 cm^–1^ was assigned to the C–H stretching vibration of alkane, while the peak at 1645.6 cm^–1^ was probably caused by polymer-bound water. Peaks were found at 1131.4, 1056.8, and 1032.6 cm^–1^, which could be attributed to C–O–C glycosidic bond vibrations and ring vibrations overlapping stretching vibrations of the group C–O–H, indicating a pyranose form of polysaccharides (Ding et al., 2012). Additionally, the α-glycosidic peak was reflected by the characteristic signal at 976.4 cm^–1^, and the α-type configuration of the mannose units was reflected by the peak near 814.3 cm^–1^.

To better capture the linkage patterns of EPS-E8, it was methylated and subjected to GC-MS. EPS-E8 contained at least 10 discernible methylated glycosidic residues (Table 2), namely 2,3,4,6-Me_4_-Man*p*, 2,4,6-Me_3_-Man*p*, 3,4,6-Me_3_-Man*p*, 2,3,4-Me_3_-Man*p*, 2,3,4-Me_3_-Glc*p*, 2,3,6-Me_3_-Glc*p*, 2,6-Me_2_-Man*p*, 2,4-Me_2_-Man*p*, 3,4-Me_2_-Man*p*, and 3,4-Me_2_-Man*p* at molar percentages of 35.53, 12.55, 20.83, 1.92, 0.96, 8.13, 0.31, 1.03, 7.49, and 11.25%, respectively. The total contents of mannose and glucose derivatives were about 79.66 and 20.34%, respectively, which is in accordance with the results of monosaccharide composition. Moreover, negative signals on linkages related to other monosaccharides (fucose, xylose, and arabinose) were obtained in this sample because of trace amounts of these monomers. Therefore, these available data confirmed that four types of corresponding glycosidic linkages, i.e., T-Man*p*-(1→ (35.53%), →3)-Man*p*-(1→ (12.55%), →2)-Man*p*-(1→ (20.83%), and →2,6)-Glc*p*-(1→ (11.24%)) were included in EPS-E8.

**TABLE 2:** Glycosidic linkage composition of methylated EPS-E8 by GC–MS analysis.

Time (min)	Methylated sugars	Deduced linkages	Molar ratios
8.85	2,3,4,6-Me_4_-Man*p*	T-Man*p*-(1→	35.53
12.33	2,4,6-Me_3_-Man*p*	→3)-Man*p*-(1→	12.55
12.41	3,4,6-Me_3_-Man*p*	→2)-Man*p*-(1→	20.83
13.63	2,3,4-Me_3_-Man*p*	→6)-Man*p*-(1→	1.92
13.72	2,3,4-Me_3_-Glc*p*	→6)-Glc*p*-(1→	0.96
14.07	2,3,6-Me_3_-Glc*p*	→4)-Glc*p*-(1→	8.13
15.24	2,6-Me_2_-Man*p*	→3,4)-Man*p*-(1→	0.31
17.93	2,4-Me_2_-Man*p*	→3,6)-Man*p*-(1→	1.03
18.25	3,4-Me_2_-Man*p*	→2,6)-Man*p*-(1→	7.50
18.25	3,4-Me_2_-Glc*p*	→2,6)-Glc*p*-(1→	11.24

### Nuclear Magnetic Resonance Spectroscopy Analysis

**FIGURE 3:**
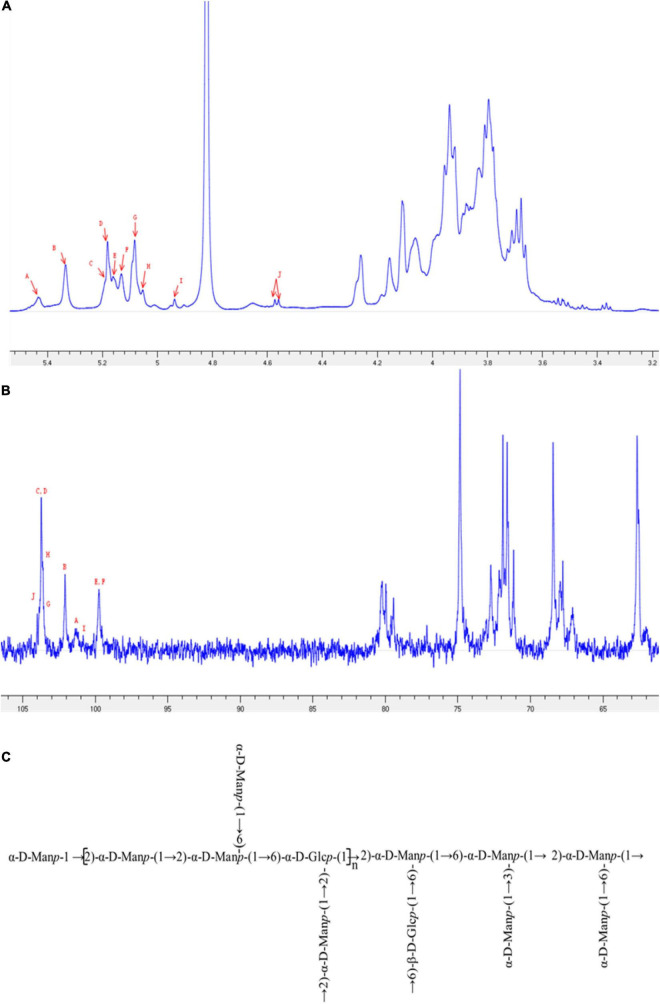
NMR spectra of EPS-E8 recorded in D_2_O at 298 K: **(A)** 1D-1H spectrum; **(B)** 1D-13C spectrum. Different signals (A–J) were defined in Table 3, respectively. **(C)** One putative structure of EPS-E8.

1D and 2D NMR spectra were used to elucidate the structural characteristics of EPS-E8. The aim was to provide information about the linkages between various monosaccharide types. In ^1^H NMR (Figure 3A) and ^13^C NMR spectra (Figure 3B), clusters of proton (H-1) and carbon (C-1) resonances was found around the region between δ 4.55–5.48 ppm and δ 99.46–104.46 ppm, respectively. The chemical shifts within δ 3.35–4.30 ppm and δ 61.04–81.25 ppm were assigned to H-2 to H-6 and C-2 to C-6 protons, respectively. EPS-E8 had 10 obvious anomeric proton signals at δ 5.42, 5.32, 5.19, 5.17, 5.15, 5.12, 5.07, 5.05, 4.93, and 4.56 ppm, labeled A–J, respectively. The remaining proton signals were corroborated by COSY and TOCSY (Supplementary Figures 2A,D), and the HSQC spectrum supported the carbon signals related to specific hydrogen signals (Supplementary Figure 2B).

In the HSQC spectrum, the chemical shift signal of anomeric residue B at δ 5.32 ppm and its correlating signal at δ 102.13 ppm were found, showing the α-configuration nature of residue B. Combined with the TOCSY spectral data, connected signals at δ 5.32/4.15, 4.15/4.01, 4.01/3.80, 3.80/3.94, and 3.94/3.79 (3.65) ppm of residue B were captured from the COSY spectrum. According to these signals, signals at δ 4.15, 4.01, 3.80, 3.94, and 3.79 (3.65) ppm were attributed to H-2 to H-6 of residue B, respectively. In combination with the HSQC spectrum, the corresponding carbon chemical shifts of C-2 to C-6 were δ 102.13, 79.37, 71.49, 67.98, 74.70, and 62.32 ppm, respectively. Notably, in comparison to previous data on mannose units, the C-2 carbon signal of 79.37 ppm shifted downward, indicating a substitution of residue B at the C-2 position. Based on these analyses, residue B was identified as →2)-α-D-Man*p*-(1→ (Wold et al., 2018; Bai et al., 2021).

Regarding the E residue, the anomeric proton chemical signal was observed at δ 5.15 ppm and its anomeric carbon at δ 99.77 ppm by HSQC spectrum. This demonstrates that residue E is an α-configuration. According to TOCSY and COSY spectra, associated signals were observed at δ 5.15/4.09, 4.09/3.79, 3.79/3.69, 3.69/3.82, and 3.82/3.92 (3.70) ppm, which was observed in the HSQC spectrum. The relative downward shifts of C-2 (δ 80.29 ppm) and C-6 carbon signals (δ 68.26 ppm) compared with published research implied that residue E was replaced at the C-2 and C-6 positions (Wang et al., 2021; Zhang et al., 2022). Therefore, residue E represented →2,6)-α-D-Man*p*-(1→. Moreover, linkages for other residues were verified using a similar approach, and the main chemical shifts of proton and carbon are listed in Table 3.

**TABLE 3:** ^1^H and ^13^C NMR chemical shift data for EPS-E8.

Sugar residue	Chemical shifts δ (ppm)						
	H-1/C-1	H-2/C-2	H-3/C-3	H-4/C-4	H-5/C-5	H-6/C-6	H-6’/C-6
A: →4)-α-D-Glc*p*-(1→	5.42/101.27	3.66/71.63	4.06/71.84	3.60/78.22	3.79/73.99	3.84/61.96	3.69
B: →2)-α-D-Man*p*-(1→	5.32/102.13	4.15/79.37	4.01/71.49	3.80/67.98	3.94/74.70	3.79/62.32	3.65
C: α-D-Man*p*-(1→	5.19/103.71	4.11/71.41	3.93/71.56	3.70/68.48	3.80/74.64	3.95/62.61	3.69
D: α-D-Man*p*-(1→	5.17/103.78	4.12/71.63	3.87/71.70	3.68/68.26	3.85/74.92	3.94/62.53	3.71
E: →2,6)-α-D-Man*p*-(1→	5.15/99.77	4.09/80.29	3.79/71.84	3.69/68.33	3.82/74.90	3.92/68.26	3.70
F: →2,6)-α-D-Glc*p*-(1→	5.12/99.63	4.06/80.15	3.82/71.79	3.68/68.05	3.84/74.78	3.92/68.19	3.69
G: →3,6)-α-D-Man*p*-(1→	5.07/103.42	4.25/71.56	4.00/79.92	3.69/68.19	3.83/74.64	3.97/67.37	3.67
H: →6)-α-D-Man*p*-(1→	5.05/103.56	4.28/71.20	3.88/71.91	3.79/68.05	3.94/74.85	3.82/68.05	3.78
I: →2,6)-β-D-Man*p*-(1→	4.93/100.99	4.04/80.07	3.82/71.84	3.72/68.08	3.81/74.89	3.82/68.49	3.68
J: →6)-β-D-Glc*p*-(1→	4.56/104.42	3.37/74.64	3.52/77.07	3.69/68.48	3.80/74.64	3.98/67.48	3.61

HMBC and NOESY spectra (Supplementary Figures 2C,E) were further employed to evaluate the correlations of these residuals and reflect the linkage positions among sugar residues. Cross-signals at δ 79.37/5.17 ppm (B C-2/D H-1), δ 71.84/5.32 ppm (E C-3/B H-1), δ 74.90/5.17 ppm (E C-5/D H-1), and δ 74.78/5.32 ppm (F C-5/B H-1) were observed in the HMBC spectrum. These crossing phenomena indicated that C-1 of residue B was correlated with H-6 and H-2 of residue F [B (1→2) F, →2)-α-D-Man*p*-(1→2,6)-α-D-Glc*p*-(1→], C-1 of residue D was linked to H-6 of residue F [D (1→2) E, α-D-Man*p*-(1→2,6)-α-D-Man*p*-(1→], and C-1 of residue D was linked to H-2 of residue B [D (1→2) B, α-D-Man*p*-(1→2)-α-D-Man*p*-(1→]. Furthermore, the correlation signals between H-1 of residue E (δ 5.12 ppm), H-3 of residue G (δ 4.00 ppm), H-1 of residue D (δ 5.17 ppm), H-2 of residue B (δ 4.15 ppm), H-1 of residue H (δ 5.05 ppm), H-2 of residue B (δ 4.15 ppm), H-1 of residue J (δ 4.56 ppm), and H-6 of residue E (δ 3.92) were obtained from the NOESY spectrum. These indicated the existence of →2,6)-α-D-Man*p*-(1→3,6)-α-D-Man*p*-(1→, α-D-Man*p*-(1→2)-α-D-Man*p*-(1→, →6)-α-D-Man*p*-(1→2)-α-D-Man*p*-(1→, and →6)-β-D-Glc*p*-(1→2,6)-α-D-Man*p*-(1→. Based on the data above, a putative structure of EPS-E8 is illustrated in Figure 3C, classifying the structure of EPS-E8 as an undocumented novel type.

### X-ray Diffraction and Thermal Stability Analysis

**FIGURE 4:**
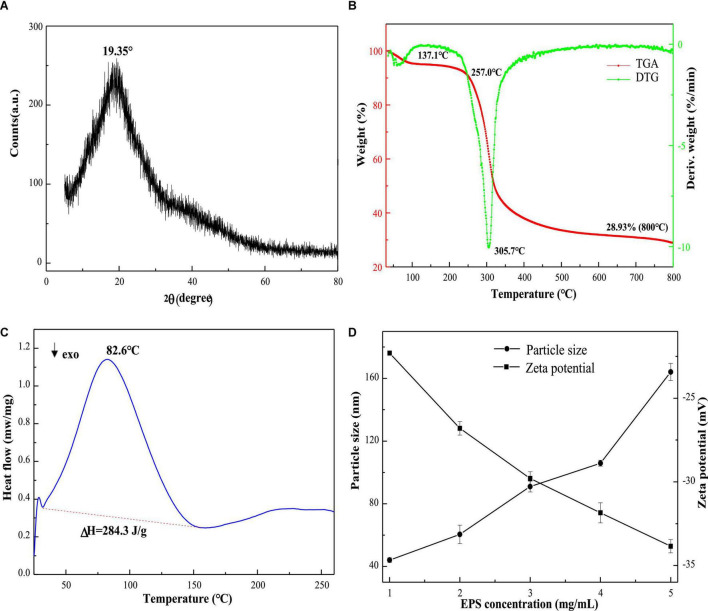
The XRD **(A)**, TGA **(B)**, and DSC **(C)** spectra of EPS-E8; the zeta potential and particle size **(D)** at various concentrations of EPS-E8.

X-ray diffraction is used to determine the crystalline degree of polysaccharides. As presented in Figure 4A, the strong peak at 19.25° indicated that the interiors of EPS-E8 have an amorphous or semi-crystalline structure. A similar profile was observed in other studies for different types of EPSs (Krishnamurthy et al., 2020; Zhao et al., 2021). This crystalline structural arrangement is known to directly affect physical properties (including solubility, emulsification, swelling power, or viscosity) of polysaccharide (Gan et al., 2020b).

The thermal properties of EPS-E8 were analyzed by TGA, and variations of thermal stability are presented in Figure 4B. The TGA curve shows two distinct mass loss stages. In the first stage, there was a mass loss of nearly 5% at 35–137.1 °C, possibly caused by the loss of polysaccharide surface-bound water molecules. The mass remained constant from 137.1 to 257 °C, suggesting that EPS-E8 is relatively stable below 257°C. The second stage of mass loss included two continuous processes within a temperature range of 257–525°C with a total mass loss of 66.1%. The first process occurred rapidly until 319°C with a mass loss of 45%, and then, the second process began at 319°C and was gradually implemented at a slow rate. The initial loss may be caused by the breakage of carbon chains and hydrogen bonds, and the slow thermal degradation may be ascribed to the production of thermally stable polysaccharides in the process. As shown in the DTG curve, the degradation temperature (Td) of EPS-E8 was 305.7°C. These results highlight the outstanding thermal stability of EPS-E8, which suggests its application potential in fields where an excessive level of thermal processing is needed.

Endothermic and exothermic processes could affect the thermal behaviors of polysaccharides associated with phase changes or deformation of the crystalline structure. As shown in Figure 4C, DSC was used to analyze the melting point and energy changes of EPS-E8 from 25 to 260°C. The melting point is an endothermic peak at about 82.6°C, and the enthalpy change (ΔH) needed to melt 1 g of EPS-E8 is 284.3 J. This result differs from previous reports on EPSs isolated from different LAB strains. The melting point of EPS-E8 is lower than that of EPS obtained from *P. pentosaceus* M41 (158.82°C) (Ayyash et al., 2020a) and *P. pentosaceus* DPS (232°C) (Abid et al., 2018). Ahmed et al. (2013) and Lobo et al. (2019) reported that the melting point and ΔH of EPS produced by *Streptococcus thermophilus* CRL1190 and *L. kefiranofaciens* ZW3 were about 74.08°C/284.46 J and 93.38°C/249.7 J, respectively. These findings strongly support the idea that the thermodynamics properties of EPS from various strains may be different.

### Particle Size and Zeta Potential

The particle size distribution and zeta potential trend of the aqueous solution of EPS-E8 are depicted in Figure 4D. As the concentration of EPS-E8 increased from 1 to 5 mg/mL, the absolute value of zeta potential increased from 22.3 to 33.85 mV. This trend indicates that the initial interfacial electric charge value and stability of the EPS-E8 aqueous solution improved with increasing concentration. Moreover, the average particle diameter of EPS-E8 in aqueous dispersion increased gradually from 44.1 to 164.1 nm with increasing concentration. The particle size of EPS-E8 is smaller than that of EPS produced by *P. pentosaceus* M41 (446.8 nm) (Ayyash et al., 2020a). The particle size may be attributed to molecular weights, types of glycosyl linkages, and monosaccharide composition. Generally, maintaining system stability demands that negatively charged surface particles strongly interact, leading to an increase in particle diameter [30]. The regular change of zeta potential and particle size confirmed the potential application of EPS-E8 in products with high sugar contents.

### Morphological Characteristics

**FIGURE 5:**
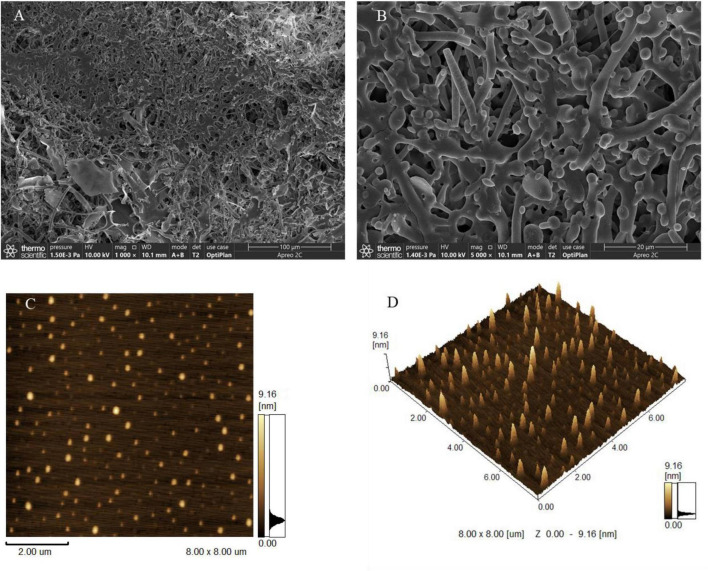
Scanning electron microscopy images of EPS-E8 under 1,000 × **(A)** and 5,000× **(B)** magnification; AFM showing the topographic features of EPS-E8, planar view **(C)** and cubic view **(D)**.

To explore the morphological characteristics of EPS-E8, SEM and AFM were applied in this study. SEM images with different magnifications (1,000× and 5,000×) are displayed in Figures 5A,B, respectively. EPS-E8 has an irregular reticular-like shape and spherical-like structure with a relatively rough surface. Interestingly, the spherical structure of EPS-E8 clearly differed from that of previously reported LAB-EPSs. For instance, EPS-C47 has a stiff-like, smooth and flake-like, and layered structure (Ayyash et al., 2020c), EPS-M41 has a compact, stiff, and layer-like structure (Ayyash et al., 2020a), while the surface of EPS-1 obtained from *Pediococcus acidilactici* MT41-11 is rough, and EPS-1 is attached to irregular shapes of differently sized blocks, and shows flaky appearance (Bai et al., 2021).

Topographical AFM images of EPS-E8 are presented in Figures 5C,D. Large amounts of spherical clusters can clearly be observed, implying molecular aggregation of polysaccharide chains. Based on these morphological characteristics, it can be predicted that side-chain hydrogen bonds play a critical role in the aggregation of polysaccharides and considerably affect the molecule conformation, functional properties, and biological activities of EPS-E8 (Hu et al., 2020).

### Emulsifying Behavior of EPS-E8

#### Emulsifying Activities With Different Oils and Hydrocarbons

**FIGURE 6:**
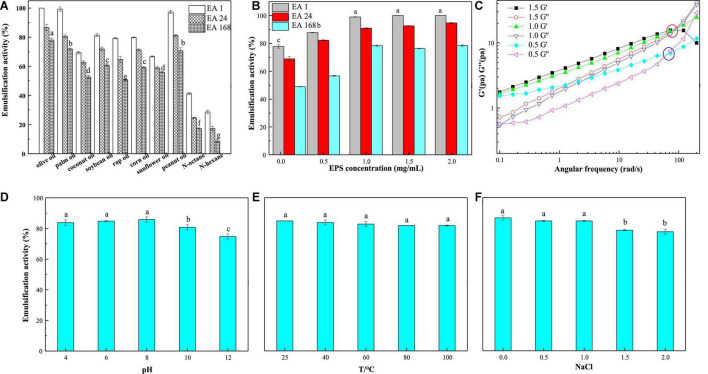
Emulsification activity of emulsions prepared with different oils (olive oil, coconut oil, peanut oil, sunflower oil, soybean oil, palm oil, rap oil, and soybean oil) and hydrocarbons (*n*-hexane and *n*-octane) **(A)** and different EPS-E8 concentrations **(B)**; oscillatory shear tests of different emulsions **(C)**; influence of pH (4.0–12.0) **(D)**, temperature (25–100°C) **(E)**, and NaCl concentration (0–2.0 M) **(F)** on the emulsification activity of the emulsions prepared with olive oil. The statistic differences were indicated with different lowercase letters (*P* < 0.05).

LAB-EPSs emulsifiers have attracted great interest owing to their excellent biocompatibility and safety. In this study, the emulsifying activities of EPS-E8 (1 mg/mL) against several edible oils and hydrocarbons were compared, and the results are shown in Figure 6A. EPS-E8 exhibited an excellent EA (100 ± 0.0%) against olive oil after 1 h. In addition, the EA values of EPS-E8 against palm oil (99.3 ± 1.5%), peanut oil (97.3 ± 1.1%), and soybean oil (81.3 ± 1.1%) were also high. Unlike the activities against tested edible oils, the EA of EPS-E8 against *n*-hexane and *n*-octane were lower, with values of 41.3 ± 0.7% and 28.7 ± 1.2%, respectively. An effective emulsifier should maintain at least 50% of the original emulsion volume 24 h after formation. According to this criterion, EPS-E8 displayed promising emulsion-stabilizing ability against all tested oils, as implied by the EA_24_ values, all of which remained much higher than 50%. However, the EA_24_ values of EPS-E8 against two hydrocarbons were lower than 50%. Notably, the EA values of EPS-E8 against all tested oils remained high even after 168 h with the lowest value of 50.7 ± 0.4% in rap oil. These results suggest that EPS-E8 has an excellent emulsifying ability against all tested oils, especially olive oil. Until now, the emulsification ability of the EPSs produced by *Pediococcus* species has not been reported. Compared to previously reported bacterial biopolymers, the emulsifying activity of EPS-E8 produced by *P. pentosaceus* E8 is promising. For instance, the EPS from *Virgibacillus salarius* BM02 showed the highest EA_24_ values of 58.82, 58.82, and 47.06% against sunflower oil, corn oil, and olive oil, respectively (Gomaa and Yousef, 2020). In research by Freitas et al. (2009), an EPS obtained from *Pseudomonas oleovorans* showed an EA_24_ value of 75% against olive oil, but EPS-E8 has a higher EA_24_ value of 86.7 ± 2.2% against olive oil at a similar concentration. These differences may be interpreted by the emulsion formation and stabilizing capacity of EPSs which is specific for certain hydrophobic compounds (Gomaa and Yousef, 2020).

#### Effect of EPS-E8 Concentration on Emulsifying Activity

To identify the probable influence of the EPS concentration on its emulsion stability against olive oil, EA values were assessed using emulsions prepared by EPS-E8 at different concentrations. Significant differences (*p* < 0.05) in the EA values were observed for EPS-E8 when the emulsion was prepared with concentrations ranging from 0 to 1 mg/mL (Figure 6B). However, no significant differences (*p* > 0.05) in EA values were observed when the EPS concentration ranged from 1 to 2 mg/mL. Therefore, 1 mg/mL is the optimal concentration for EPS-E8 when it is used as emulsifier as there is no significant improvement in its emulsifying ability at higher concentrations. Similar findings were reported with EPS derived from other bacterial species. Han et al. (2015) reported that 1 mg/mL was the optimal concentration of EPS produced by *Bacillus amyloliquefaciens* LPL061 in emulsifying sunflower seed oil. In another study, 1 mg/mL of EPSs from *Bifidobacterium longum* subsp. *infantis* CCUG 52486 and *Bifidobacterium infantis* NCIMB 702205 effectively produced an emulsion with sunflower seed oil (Prasanna et al., 2012).

The stability of emulsions can be determined using small amplitude oscillatory shear tests. The results of olive oil-in-water emulsions stabilized with EPS at concentrations of 0.5, 1, and 1.5% (w/v) are shown in Figure 6C. The storage modulus values (G′) and loss modulus values (G″) of emulsions increased with increasing angular frequency, and G′ values exceeded G″ values at low frequencies. Thus, the emulsions showed a solid-like viscoelastic behavior. The curves of G′ and G″ had crossover points as the angular frequency increased. This phenomenon showed that emulsions are typical entangled polymer solutions. In addition, the crossover points of G′ and G″ moved rearward with increasing EPS-E8 concentration because of the polysaccharide network that formed around the dispersed phase droplet (Lopez-Ortega et al., 2020). Hence, a higher concentration of EPS in the emulsion can enhance the formation of a network structure and therefore, improve the physical stability of emulsions.

#### Effects of pH, Temperature, and Ionic Strength on Emulsion Stability

A challenge for most industries is to retain emulsifier activity when subjected to extreme physicochemical conditions, namely pH, temperature, and ionic strength. Therefore, the emulsion forming and stabilizing capacities of EPS-E8 were assessed for different EPS-E8 concentrations (0–2 mg/mL), temperatures (25–100 °C), pH (4–12), and NaCl concentrations (0–2 M). The results are shown in Figure 6D. The emulsions of EPS-E8 with olive oil were only stable in acidic and neutral conditions; however, a significant decrease in the EA was observed in alkaline conditions (pH 10 and 12). Similar stabilities have been reported for EPS produced by *Pseudomonas stutzeri* AS22 (Maalej et al., 2016). However, EPS-E8 was thermally stable and retained its emulsifying activity at temperatures of up to 100°C (Figure 6E). This contrasted with previously reported studies, wherein the emulsifying activity of certain natural polymers decreased at high temperatures, such as the emulsion prepared using EPS from *Virgibacillus salarius* BM02 with sunflower oil, which decreased to 47.06% at 100°C (Gomaa and Yousef, 2020). The EPS produced by haloarchaeon *Haloferax mucosum* was shown to hold good emulsifying stability with n-hexane, within a temperature range of 25–100°C (Lopez-Ortega et al., 2020). These results indicate that the emulsion stability, at high temperatures, can be attributed to the utilized emulsifier, rather than to the employed hydrophobic compound, which may be explained by the thermal stability of EPS-E8 of up to 305.7°C (Maalej et al., 2016). Additionally, the emulsion stability was assessed under different salinities (0–2 M NaCl). EPS-E8 stabilized emulsions in combination with NaCl, showing less than 15% reduction in the initial EA_24_ values when the NaCl concentration reached up to 2 M (Figure 6F). According to these results, EPS-E8 might find potential applications in food, cosmetic, and detergent fields as an emulsifier.

### Antioxidant Activities of Exopolysaccharide

**FIGURE 7:**
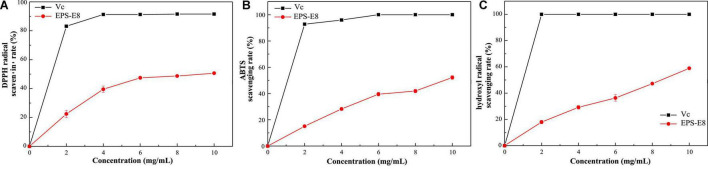
The antioxidant activities of EPS-E8 at different concentrations using VC as a control: Scavenging activity toward DPPH **(A)**; ABTS radical **(B)**; Hydroxyl radical **(C)** of EPS-E8.

Exopolysaccharide have been widely explored for their antioxidant capacities, and their antioxidant activity can be assessed based on various mechanisms and reactions (Jeddou et al., 2016). In this study, the *in vitro* antioxidant activity of EPS-E8 was explored by measuring its DPPH (Figure 7A), ABTS (Figure 7B), and hydroxyl radical (Figure 7C) scavenging activities. Within the tested EPS-E8 concentrations (0–10 mg/ml), the antioxidant capacities of EPS-E8 showed a positive correlation with its concentrations, but the antioxidant capacities of EPS-E8 were significantly lower than those of the control VC (*P* < 0.05). When the EPS concentration increased to 10 mg/mL, the scavenging activities of EPS-E8 towards DPPH, ABTS, and hydroxyl radical increased to 50.62 ± 0.5%, 52.17 ± 1.4%, and 58.91 ± 0.7%, respectively. The DPPH radical scavenging value exceeds those of EPSs produced by *L. plantarum* YW32 (30%) (Ji et al., 2015) and *L. plantarum* KX041 (37.48%) (Xu et al., 2019). These results demonstrate the good antioxidant activity of EPS-E8 and its further exploration as an antioxidant is warranted.

### Effect of Exopolysaccharide on the Preservation of Strawberry

#### Weight Loss Ratio

**FIGURE 8:**
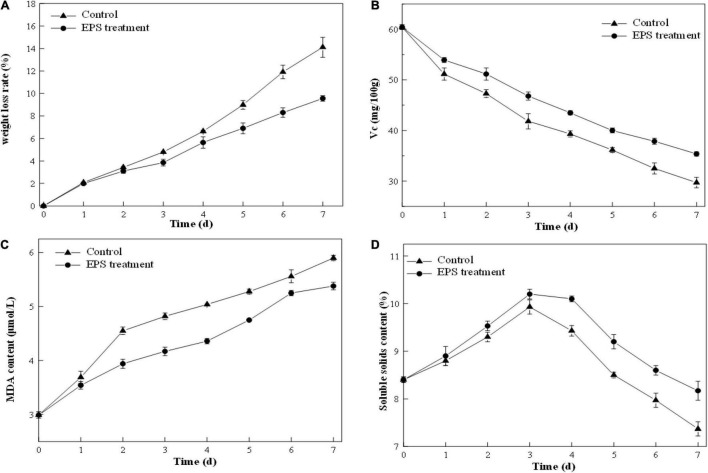
Postharvest qualities of strawberry fruit: Weight loss ratio **(A)**, vitamin C content **(B)**, malondialdehyde content **(C)**, soluble solids content **(D)** of strawberry fruit coated with EPS-E8 and uncoated control fruit.

The weight loss ratio of strawberry is an important parameter to reflect the respiration rate and moisture evaporation during preservation. As illustrated in Figure 8A, both control and EPS-E8-treated samples revealed an increased weight loss ratio along with the storage. However, the increase in the weight loss ratio of EPS-E8-treated samples was slower than that of the control samples. On the third day, the weight loss ratio of EPS-E8-treated samples reached 3.85 ± 0.3%, yet was still significantly lower than that of the control (4.80 ± 0.03%) (*p* < 0.05). The control reached the highest weight loss ratio value of 14.11 ± 0.89% on the seventh day, while samples treated with EPS-E8 showed a value of 9.56 ± 0.24% (*p* < 0.05). Xu and Wu (2021) reported that in mango fruits treated with fucoidan coating, the weight loss rates were lower than that of the control group. Therefore, our results suggest that the exploited exopolysaccharide-based coating forms a protective film to decrease respiration and transpiration rates, and thus delaying senescence while preserving quality of fruits (Guerreiro et al., 2015).

#### Vitamin C Content

A decline trend of VC content happens in fruit senescence, and it shows a weakened antioxidant activity of fruit. According to Figure 8B, the VC content of the control and EPS-E8-treated samples decreased gradually with the extension of storage period. However, the VC content of EPS-E8-treated strawberries was much higher than that of the control during storage (*p* < 0.05). The VC content of EPS-E8-treated strawberries reached its minimum value (35.37 ± 0.44 mg/100 g) on the seventh day but was still higher than that of the control group (29.71 ± 1.03 mg/100 g) (*p* < 0.05). This result suggested that EPS-E8 could effectively delay the senescence of strawberries. In a similar study, Hong et al. (2012) found that a polysaccharide-based coating effectively retarded the VC loss of guava. This could be attributed to EPS coating preventing the exchange of gasses between the inside and outside of the fruit and decelerating the oxidation of VC into dehydrogenated ascorbic acid (Mditshwa et al., 2017).

#### Malondialdehyde Content

Malondialdehyde is an end-product of lipid peroxidation of membranes and is considered an important indicator of the integrity and freshness of strawberry cell membranes (Saleem et al., 2021). As indicated in Figure 8C, the MDA content continuously increased during the storage period. However, the MDA content was significantly lower (*P* < 0.05) in EPS-E8-treated strawberries in comparison with the control group. This result was in agreement with the study conducted by Yuan et al. (2020), who reported that treatment with polysaccharides from *Pythium arrhenomanes* resulted in a lower MDA content than the control group during the storage of strawberry. The influence of EPS-E8 treatment on the MDA content of strawberries was probably caused by the deceleration of respiration and metabolism, thereby decelerating the maturation process. Thus, EPS-E8 can reduce the lipid peroxidation of cell membranes and alleviate tissue damage in fruits to some extent.

#### Soluble Solids

The soluble solids can mirror the maturity of fruits and represent the taste since they are mostly constituted of sugar and organic acids. The change of soluble solid content in all samples during storage is shown in Figure 8D. The soluble solids in the control and EPS-E8-treated strawberries reached the highest values (10.20 ± 0.10 and 9.93 ± 0.15, respectively) at the third day. This phenomenon might be caused by the original metabolic process that converts carbohydrates into sugars and other soluble compounds (Lan et al., 2019). After this period of increase, the soluble solids content decreased rapidly in the following four days, probably due to the quick consumption of soluble solids during respiration. However, the decrease in soluble solids contents of EPS-E8-treated samples was slower than that of the control samples. Thus, the utilization of EPS-E8 reduced the degradation of soluble solids and was beneficial to maintaining a better level of soluble solids. Our results are in line with Tahir et al. (2018), who determined that the lower respiration rates of fruit coated with polysaccharide-based may be ascribed to the preservation of higher carbohydrates in the fruits. Thus, it can be speculated that EPS-E8 might find application as a polysaccharide-based coating to improve the shelf-life of strawberries.

## Conclusion

In this study, a novel EPS (EPS-E8), produced by *P. pentosaceus* E8, was isolated and characterized. EPS-E8 is a heteropolysaccharide with a molecular weight of 5.02 × 10^4^ g/mol, possesses a main chain of →2)-α-D-Man*p*- (1→2,6)-α-D-Glc*p*-(1→6)-α-D-Man*p*-(1→, and branches with α-D-Man*p*-(1→2)-α-D-Man*p*-(1→ and α-D-Man*p*-(1→. EPS-E8 is a semicrystalline biopolymer with favorable thermal stability that has an irregular reticular-like shape when observed at magnification. The initial interfacial electric charge value and average particle diameter of EPS-E8 aqueous solution improved with increasing concentration. EPS-E8 exhibits significant antioxidant potential. In addition, EPS-E8 exhibits good emulsifying properties against various tested substrates, except for *n*-hexane and *n*-octane. The excellent stability of the emulsifying activity of the EPS-E8 at extreme physicochemical conditions, suggests it as a promising emulsifier candidate in the food, pharmaceutical, and cosmetic industries, as well as for the biotreatment of hydrocarbon-polluted environments. In addition, when EPS-E8 was tested for its effects in strawberry preservation, the results obtained collectively revealed that EPS-E8 may be developed as a polysaccharide-based coating to extend the shelf-life of strawberry in the near future.

## Data Availability Statement

The Data presented in this study are deposition in the NCBI repository, accession number: ok483363.

## Author Contributions

GJ: conceptualization, investigation, and writing – original draft preparation. JH: software. LG: methodology. XL: validation and data curation. ZX and LY: formal analysis and software. RL: revision. YT: conceptualization, funding acquisition, supervision, writing – reviewing and editing, and project administration. All authors contributed to the article and approved the submitted version.

## Conflict of Interest

The authors declare that the research was conducted in the absence of any commercial or financial relationships that could be construed as a potential conflict of interest.

## Publisher’s Note

All claims expressed in this article are solely those of the authors and do not necessarily represent those of their affiliated organizations, or those of the publisher, the editors and the reviewers. Any product that may be evaluated in this article, or claim that may be made by its manufacturer, is not guaranteed or endorsed by the publisher.
