# Supplementary material for: Exopolysaccharide Produced by Pediococcus pentosaceus E8: Structure, Bio-Activities, and Its Potential Application
Source: Front Microbiol. 2022 Jun 22;13:923522. doi: 10.3389/fmicb.2022.923522 (PMC9257109; doi:10.3389/fmicb.2022.923522)

**Fig. S1** SEM image of *Pediococcus pentosaceus* E8


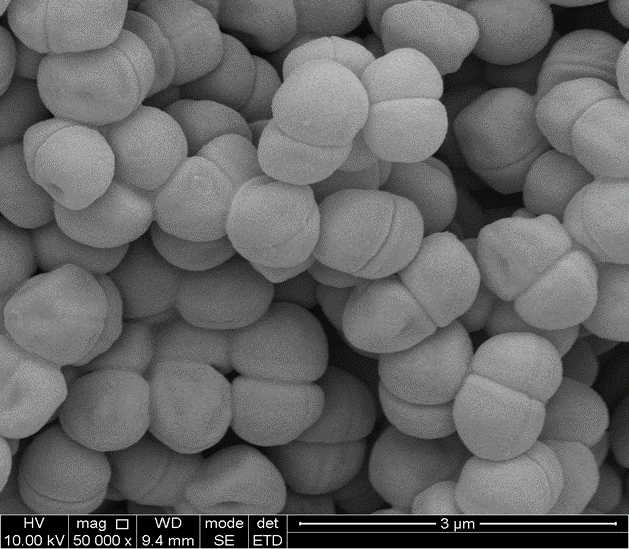


**Fig. S2** NMR spectra of EPS-E8 recorded in D_2_O at 298 K: (A) COSY spectrum; (B) HSQC spectrum; (C) HMBC spectrum; (D) TOCSY spectrum; (E) NOESY spectrum.


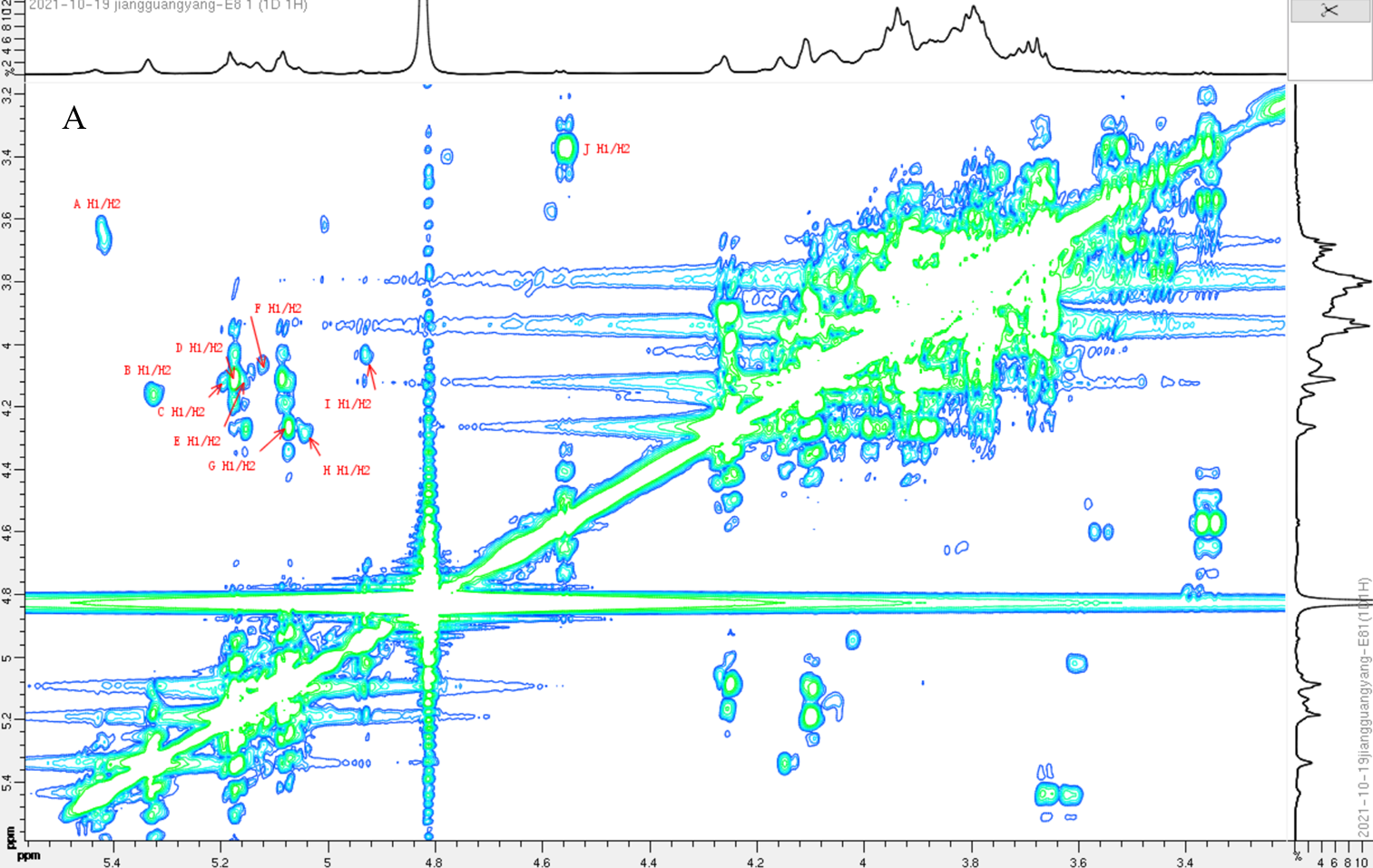


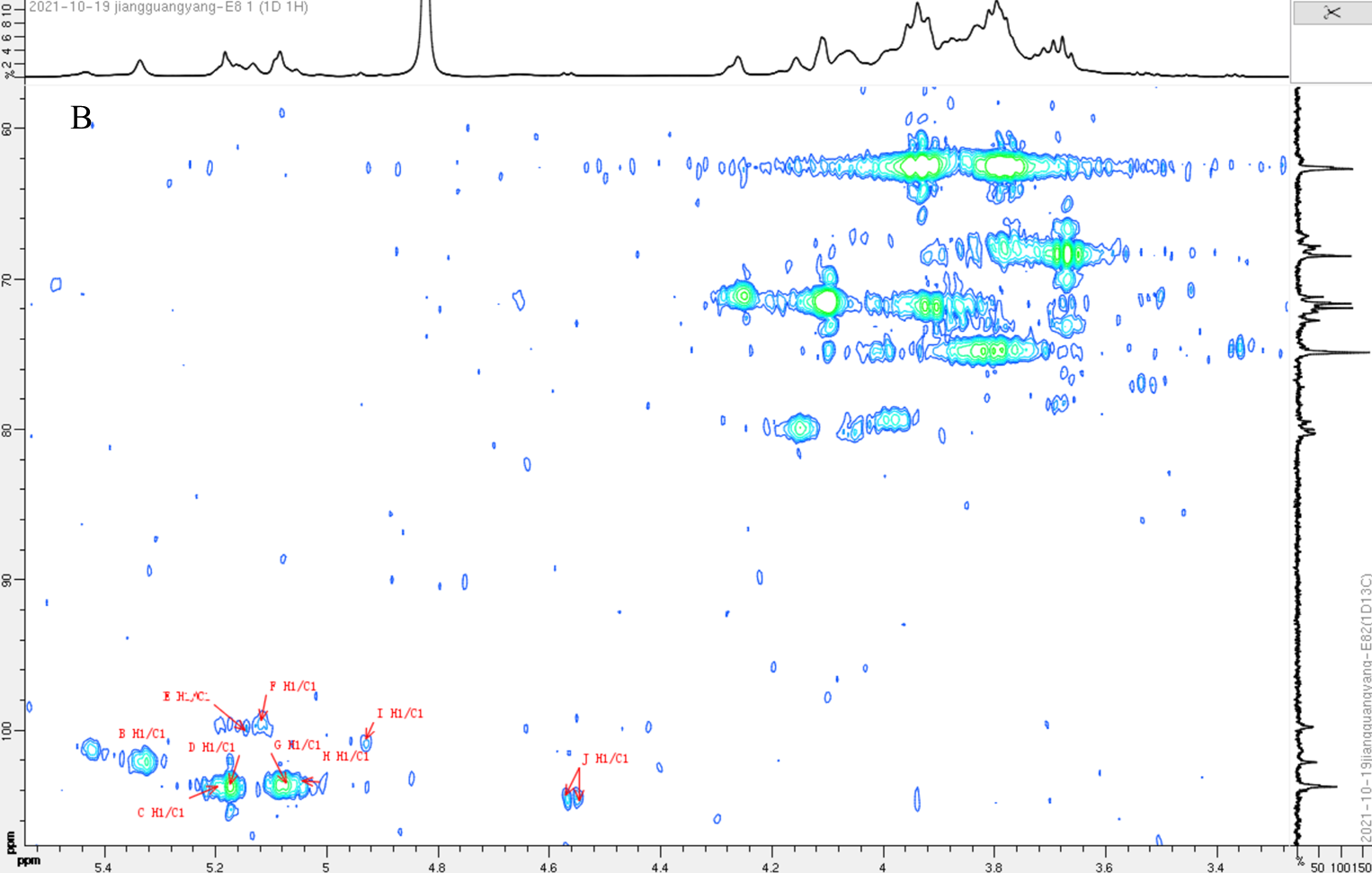


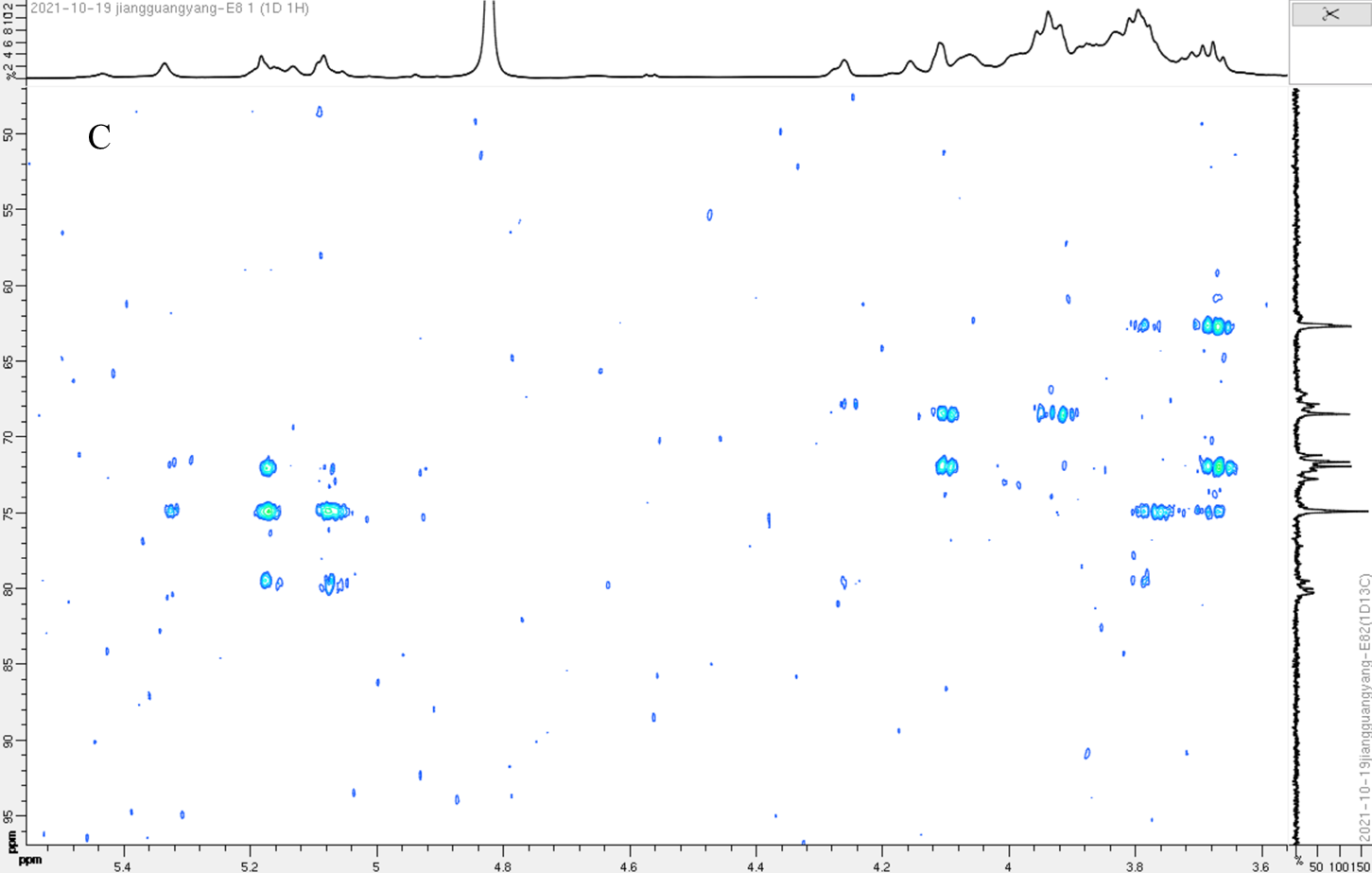


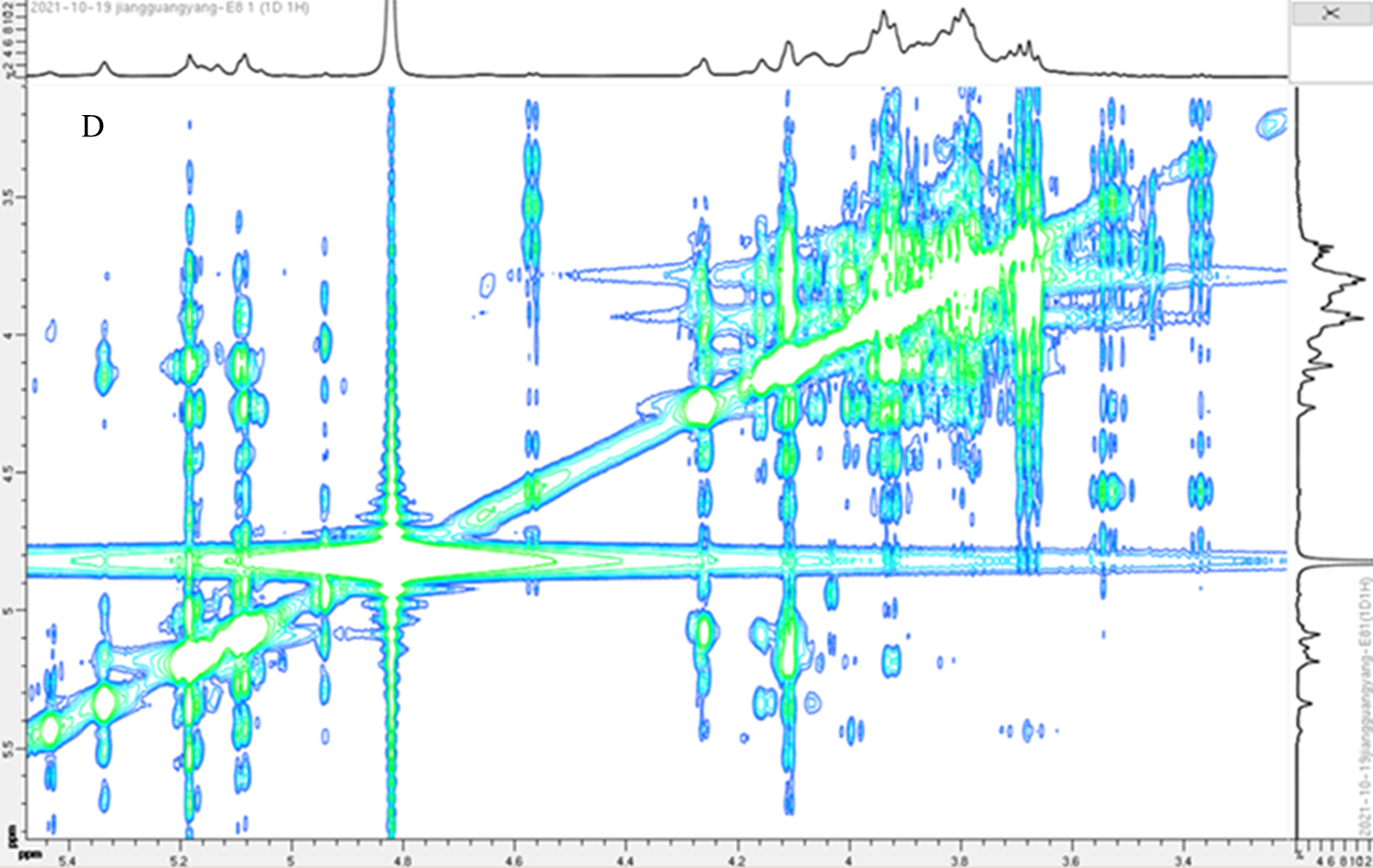


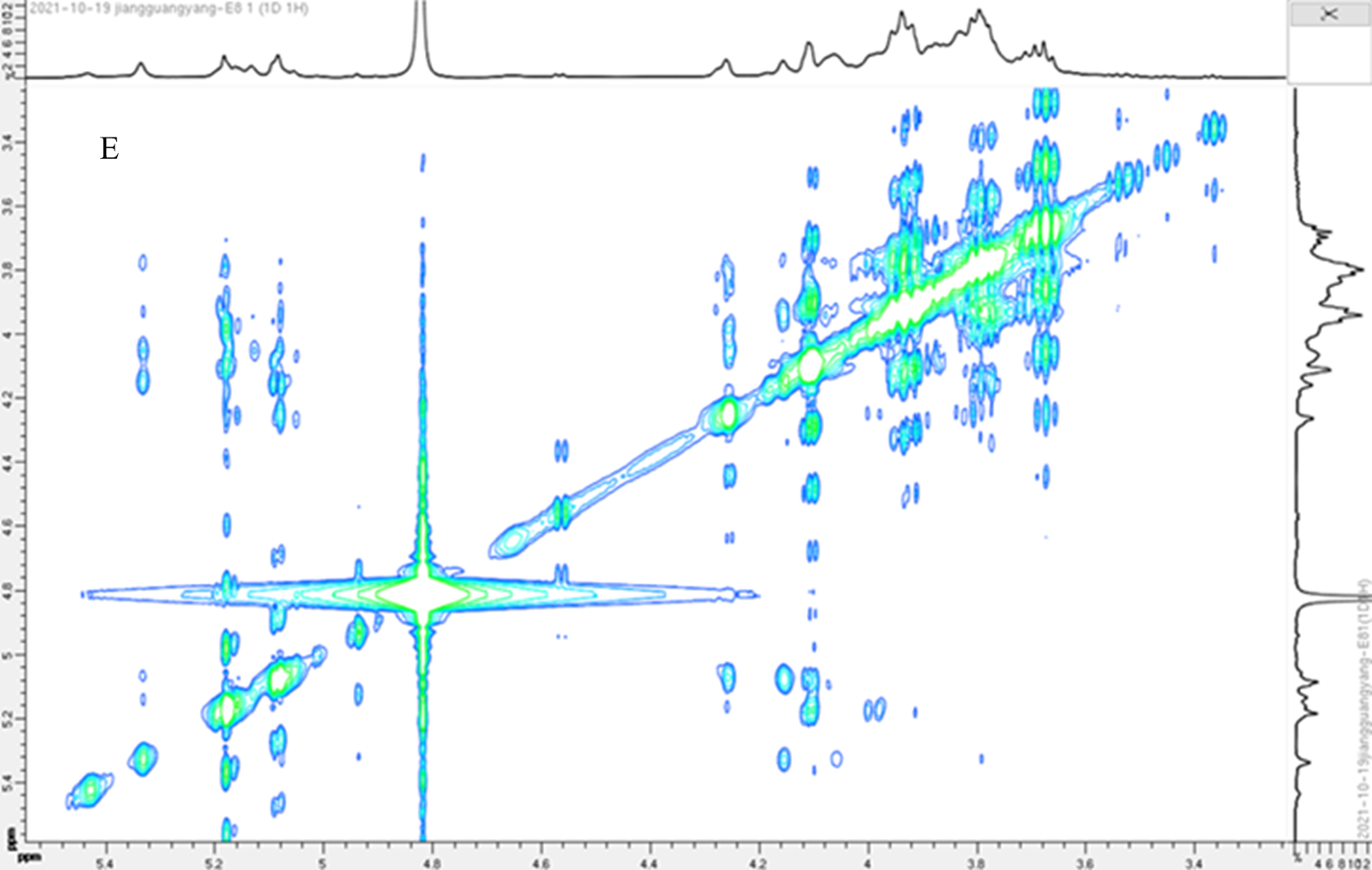

Supplement: Supplementary file 1 [file Data_Sheet_1.DOCX]
